# Supplementary material for: Mutational biases and selection in mitochondrial genomes: insights from a comparative analysis of natural and laboratory populations of Caenorhabditis elegans
Source: G3 (Bethesda). 2025 Dec 29;16(3):jkaf310. doi: 10.1093/g3journal/jkaf310 (PMC12958816; doi:10.1093/g3journal/jkaf310)
Supplement: jkaf310_Supplementary_Data [file jkaf310_supplementary_data.zip › Schifano_et_al-SupplMaterial.pdf]

Supplementary Material

Schifano *et al.* *"Mutational Biases and Selection in Mitochondrial Genomes: Insights from a Comparative Analysis of Natural and Laboratory Populations of Caenorhabditis elegans."*

Supplementary Table 1. Comparisons of observed and expected counts of synonymous polymorphisms with flanking G/C or A/T bases. Synonymous polymorphisms were analyzed separately for two-fold and four-fold degenerate sites.

Codon degeneracy	Neighbor direction	Neighboring nucleotide	Observed counts	Expected counts	*G*-test statistic	*p*-value
**Two-fold**	**5′**	**A/T**	729	773.43	29.27	6.3×10^{-8} **
		**G/C**	105	60.57		
	**3′**	**A/T**	566	640.80	34.91	3.5×10^{-9} **
		**G/C**	268	193.20		
**Four-fold**	**5′**	**A/T**	161	243.28	41.54	1.2×10^{-10} **
		**G/C**	739	656.72		
	**3′**	**A/T**	485	621.56	90.60	$< 2.2 \times 10^{-16}$ **
		**G/C**	415	278.44		

Supplementary Table 2. McDonald-Kreitman test-statistics and associated significance level for each of the 12 protein-coding genes of the *C. elegans* mtDNA genome. The notations d_N and d_S represent fixed nonsynonymous and synonymous difference between *C. elegans* and *C. brenneri*, respectively, and p_N and p_S represent polymorphic nonsynonymous and synonymous changes within the *C. elegans* natural isolates.

Gene	d_S	d_N	p_S	p_N	Neutrality index	*p*-value; Fisher's exact test
nd6	44	23	31	11	0.68	0.40
nd4L	12	5	10	2	0.48	0.66
nd1	78	18	61	9	0.64	0.40
atp6	52	11	34	3	0.42	0.24
nd2	76	23	40	14	1.16	0.70
ctb1	93	17	72	10	0.76	0.68
cox3	75	1	46	2	3.26	0.56
nd4	113	14	72	12	1.35	0.52
cox1	121	7	117	5	0.74	0.77
cox2	55	5	44	4	1.00	1.00
nd3	27	4	17	2	0.79	1.00
nd5	143	21	81	9	0.76	0.55
**Total**	889	149	625	83	0.79	0.11

Supplementary Table 3. Comparison of the base distribution at four-fold degenerate sites between codon families. *Ad-hoc* tests were performed comparing the base composition at synonymous sites for individual codon families to four-fold degenerate sites in other codon families. The *p-value* threshold for *ad-hoc* tests is 0.05/7 = 0.0071. The Pearson's χ^2 was calculated from 10,000 simulations because some bases at the 3rd codon position were present in fewer than five codons per amino acid.

Amino Acid	T	A	G	C	Pearson's χ^2 test statistic (*p*-value)
**Alanine**	62	39	4	9	16.59 (0.0014*)
**Glycine**	93	60	29	5	16.89 (0.0018*)
**Proline**	27	39	8	7	8.24 (0.0399)
**Serine 1**	61	126	39	6	57.98 (9.99e-05**)
**Serine 2**	84	51	5	5	18.97 (0.0008*)
**Threonine**	62	69	6	7	8.36 (0.0370)
**Valine**	118	98	28	9	2.19 (0.5365)
**TOTAL**	507	482	119	48	90.74 (9.99e-05**)

Supplementary Table 4. Comparison of base distribution at two-fold degenerate sites between codon families ending in T or C bases. *Ad-hoc* tests were performed comparing the base composition at synonymous sites for individual codon families to two-fold degenerate sites in other codon families ending in T or C bases. The *p-value* threshold for *ad-hoc* tests is 0.05/7 = 0.0071. The Pearson's χ^2 was calculated from 10,000 simulations because some bases at the 3rd codon position were present in fewer than five codons per amino acid.

Amino Acid	T	C	Pearson's χ^2 test statistic (*p*-value)
**Cysteine**	45	2	1.37 (0.3170)
**Aspartic Acid**	55	8	1.20 (0.3763)
**Phenylalanine**	429	23	17.27 (9.99e-05*)
**Histidine**	45	13	15.11 (0.0012*)
**Isoleucine**	257	23	0.34 (0.6055)
**Asparagine**	139	12	0.26 (0.6721)
**Tyrosine**	144	29	19.95 (2e-04*)
**TOTAL**	1,114	110	36.72 (9.99e-05**)

Supplementary Table 5. Comparison of base distribution at two-fold degenerate sites between codon families ending in A or G bases. *Ad-hoc* tests were performed comparing the base composition at synonymous sites for individual codon families to two-fold degenerate sites in other codon families ending in A or G bases. The *p-value* threshold for *ad-hoc* tests is 0.05/6 = 0.0083. The Pearson's χ^2 was calculated from 10,000 simulations because some bases at the 3rd codon position were present in fewer than five codons per amino acid.

Amino Acid	A	G	Pearson's χ^2 test statistic (*p*-value)
**STOP**	10	2	1.35 (0.2362)
**Glutamic Acid**	52	26	63.99 (9.99e-05*)
**Lysine**	95	14	1.71 (0.2424)
**Methionine**	134	44	56.39 (9.99e-05*)
**Glutamine**	34	10	5.78 (0.0251)
**Tryptophan**	62	9	1.07 (0.2936)
**TOTAL**	387	105	15.96 (0.0072**)

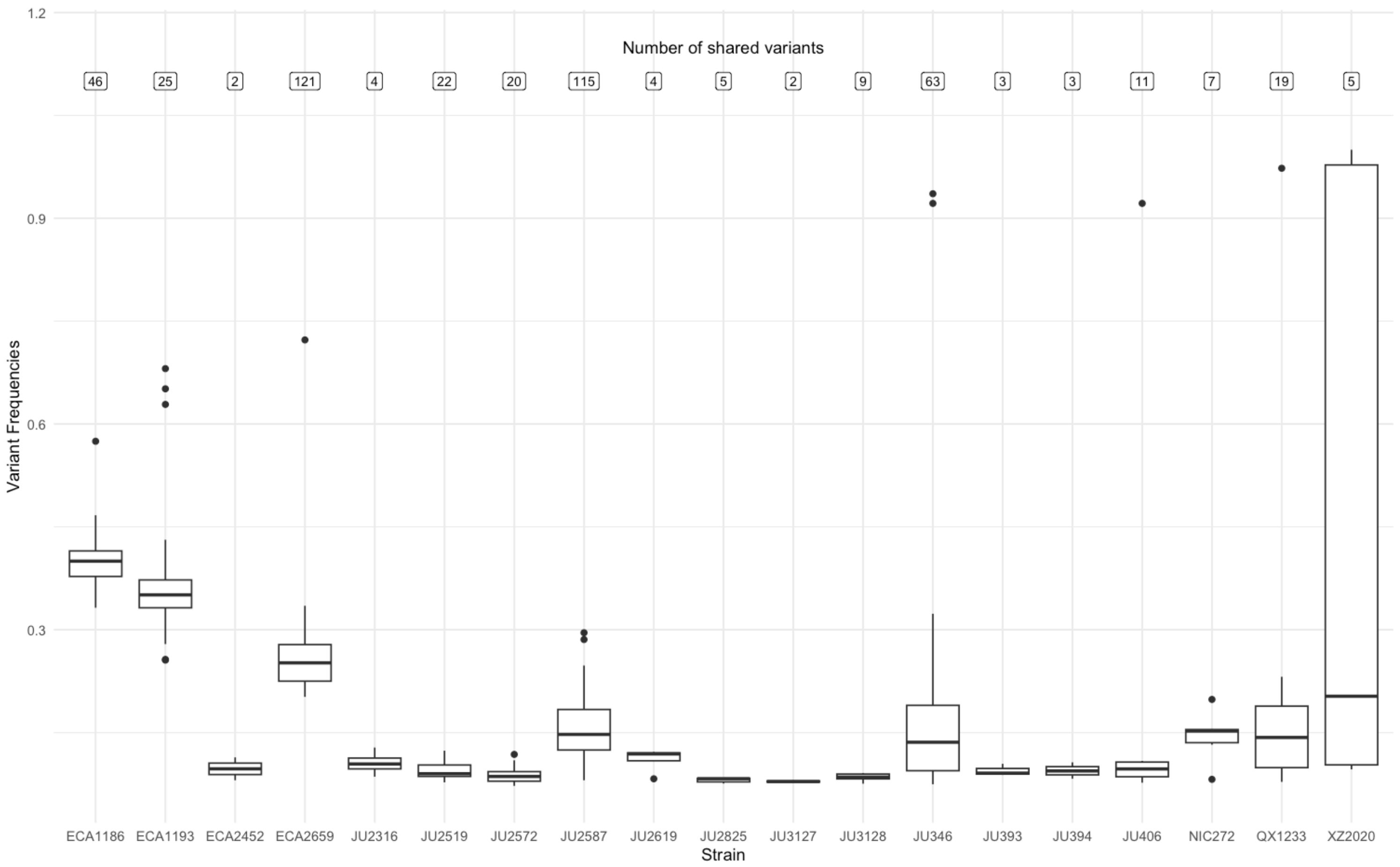

Supplementary Fig. 1. The frequencies of mtDNA variant calls in 19 *C. elegans* natural isolates from the CaeNDR database (Crombie et al. 2024) with >1 variant call per line in low frequency that were also shared with fixed variants in other natural isolates. The top of the figure lists the number of shared variants per line that are not fixed. We suspect that these calls did not result from true heteroplasmic variants but from DNA contamination from other natural isolates. Data from these lines was not included in the final analysis of the substitution spectrum in natural isolates.

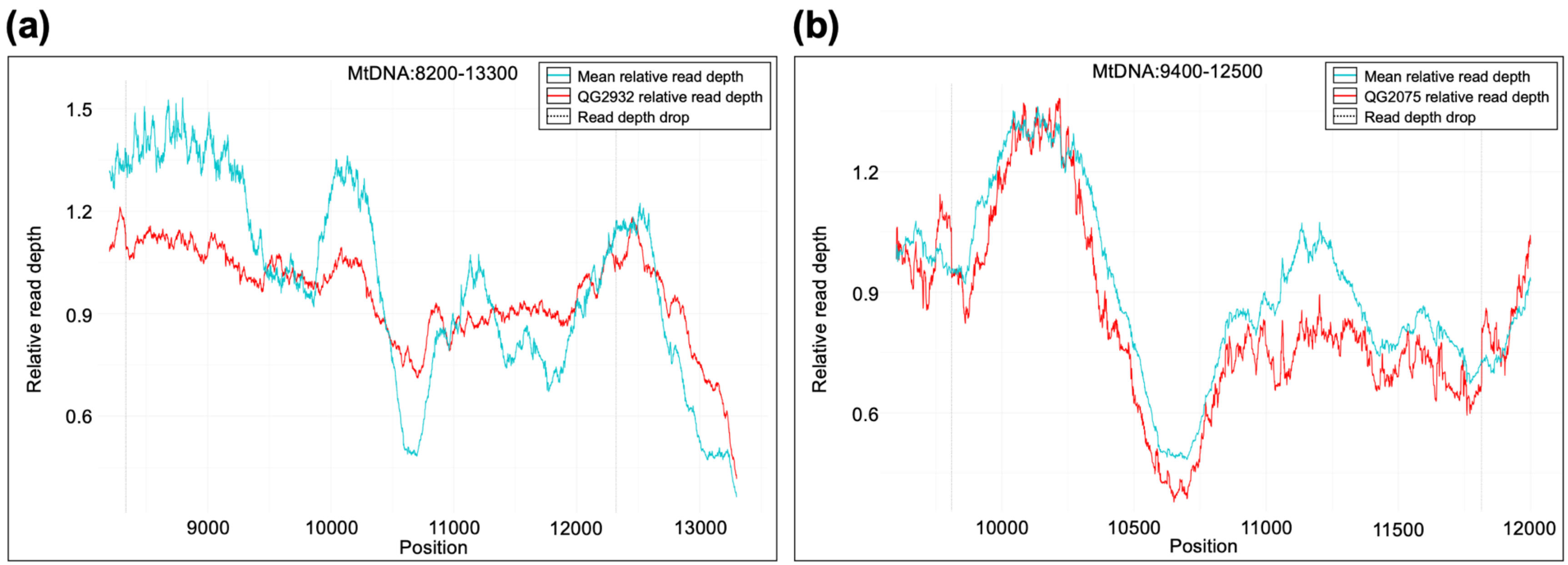

Supplementary Fig. 2. The distribution of read depth for two mitochondrial genomes with low heteroplasmic frequencies of mtDNA deletions. The horizontal axis represents the position in the reference strain mtDNA molecule and the vertical axis represents the relative read depth at any given position. The red and blue line is the distribution of relative read depth in the mtDNA harboring the deletion and mtDNA in all other natural isolates, respectively. The two grey vertical lines indicate the position of the deletion breakpoints which were identified independently in Delly, Manta and Pindel. a) Deletion in natural isolate QG2932. b) Deletion in natural isolate QG2075.

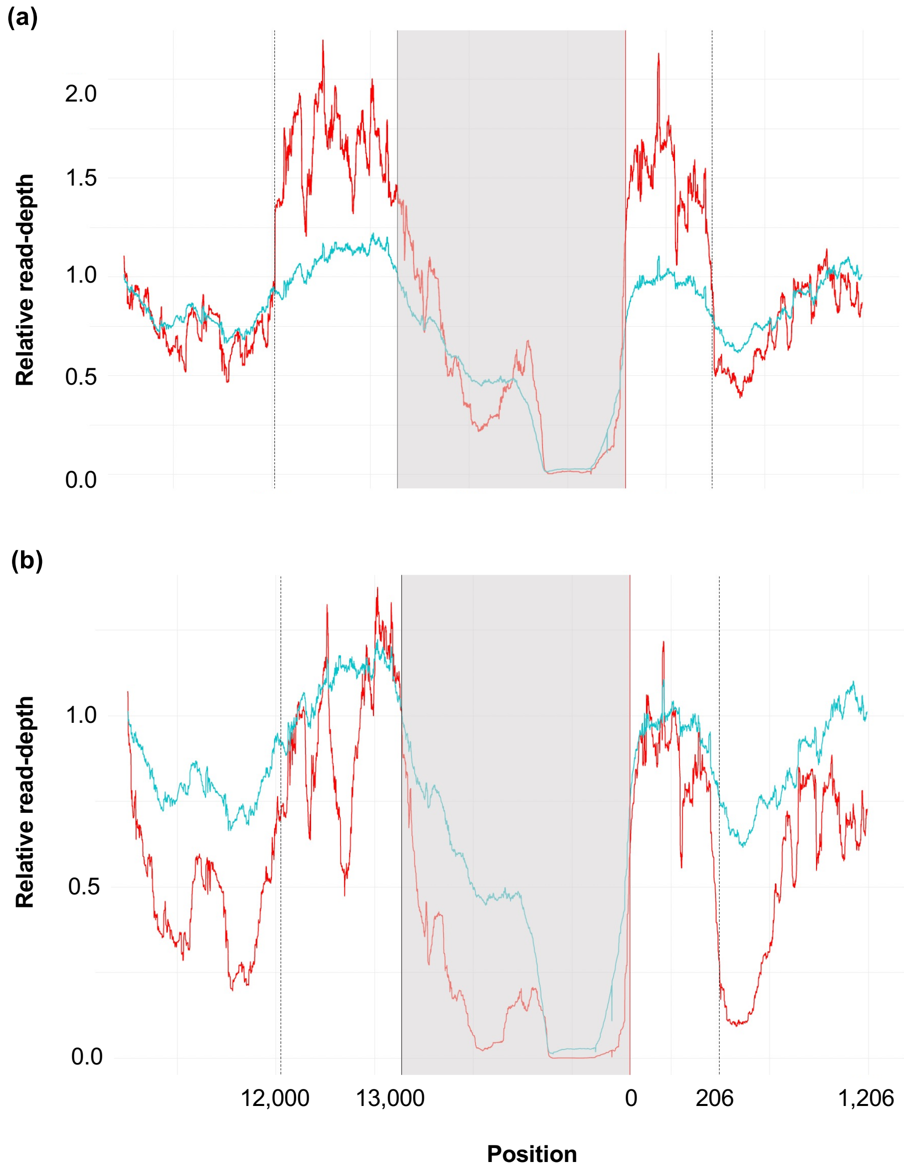

Supplementary Fig. 3. The distribution of read depth for two mitochondrial genomes with low heteroplasmic frequencies of mtDNA duplications. The breakpoints at the 5′ and 3′ ends of the duplications are shown in separate panels. The horizontal axis represents the position in the reference strain mtDNA molecule and the vertical axis represents the relative read depth at any given position. The red and blue lines represent the distribution of relative read depth in the mtDNA harboring the duplication and mtDNA in all other natural isolates, respectively. The two grey vertical lines indicate the position of the duplication breakpoints which were identified independently in Delly, Manta and Pindel. a) The left and right panels represent the 5′ and 3′ breakpoint of the duplication identified in natural isolate PS2025, respectively. b) The left and right panels represent the 5′ and 3′ breakpoint of the duplication identified in natural isolate MY25, respectively.
