# Supplementary figures and images for: Mutational biases and selection in mitochondrial genomes: insights from a comparative analysis of natural and laboratory populations of Caenorhabditis elegans
Source: G3 (Bethesda). 2025 Dec 29;16(3):jkaf310. doi: 10.1093/g3journal/jkaf310 (PMC12958816; doi:10.1093/g3journal/jkaf310)

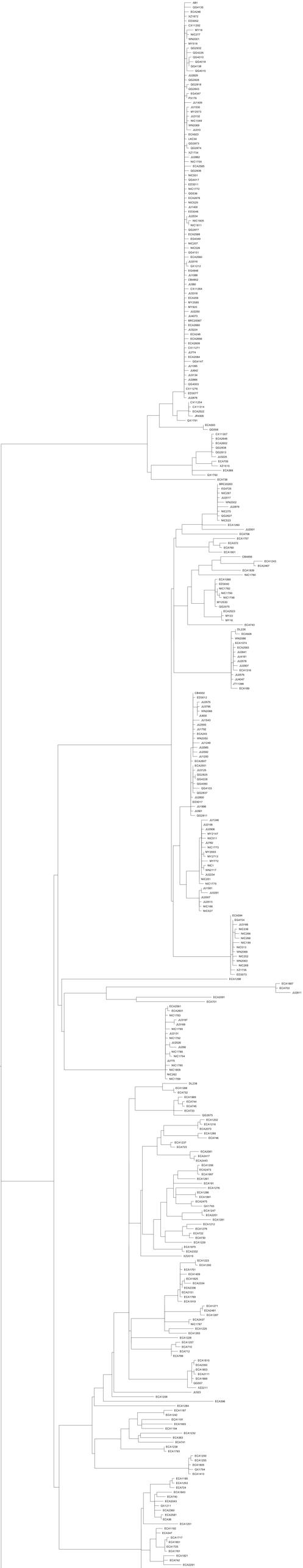

Supplement: jkaf310_Supplementary_Data [file jkaf310_supplementary_data.zip › SupplFile2.pdf]
